# Synergistic effects of the immune checkpoint inhibitor CTLA-4 combined with the growth inhibitor lycorine in a mouse model of renal cell carcinoma

**DOI:** 10.18632/oncotarget.15505

**Published:** 2017-02-19

**Authors:** Xiezhao Li, Peng Xu, Chongshan Wang, Naijin Xu, Abai Xu, Yawen Xu, Takuya Sadahira, Motoo Araki, Koichiro Wada, Eiji Matsuura, Masami Watanabe, Junxia Zheng, Pinghua Sun, Peng Huang, Yasutomo Nasu, Chunxiao Liu

**Affiliations:** ^1^ Department of Urology, Zhujiang Hospital, Southern Medical University, Guangzhou, China; ^2^ Department of Urology, Okayama University Graduate School of Medicine, Dentistry and Pharmaceutical Sciences, Okayama, Japan; ^3^ Okayama Medical Innovation Center, Okayama University, Okayama, Japan; ^4^ Center for Innovative Clinical Medicine, Okayama University Hospital, Okayama, Japan; ^5^ Faculty of Chemical Engineering and Light Industry, Guangdong University of Technology, Guangzhou, China; ^6^ Institute of Traditional Chinese Medicine and Natural Products, College of Pharmacy, Jinan University, Guangzhou, China

**Keywords:** renal cell carcinoma, anti-CTLA-4, lycorine, immunotherapy, preclinical model

## Abstract

Renal cell carcinoma (RCC) management has undergone a major transformation over the past decade; immune checkpoint inhibitors are currently undergoing clinical trials and show promising results. However, the effectiveness of immune checkpoint inhibitors in patients with metastatic RCC (mRCC) is still limited. Lycorine, an alkaloid extracted from plants of the Amaryllidaceae family, is touted as a potential anti-cancer drug because of its demonstrative growth inhibition capacity (induction of cell cycle arrest and inhibition of vasculogenic mimicry formation). Moreover, T cell checkpoint blockade therapy with antibodies targeting cytotoxic T-lymphocyte associated protein 4 (CTLA-4) has improved outcomes in cancer patients. However, the anti-tumor efficacy of combined lycorine and anti-CTLA-4 therapy remains unknown. Thus, we investigated a combination therapy of lycorine hydrochloride and anti-CTLA-4 using a murine RCC model. As a means of *in vitro* confirmation, we found that lycorine hydrochloride inhibited the viability of various RCC cell lines. Furthermore, luciferase-expressing Renca cells were implanted in the left kidney and the lung of BALB/c mice to develop a RCC metastatic mouse model. Lycorine hydrochloride and anti-CTLA-4 synergistically decreased tumor weight, lung metastasis, and luciferin-staining in tumor images. Importantly, the observed anti-tumor effects of this combination were dependent on significantly suppressing regulatory T cells while upregulating effector T cells; a decrease in regulatory T cells by 31.43% but an increase in effector T cells by 31.59% were observed in the combination group compared with those in the control group). We suggest that a combination of lycorine hydrochloride and anti-CTLA-4 is a viable therapeutic option for RCC patients.

## INTRODUCTION

Renal cell carcinoma (RCC) is the twelfth most common cancer in the world. In the United States, approximately 62,700 new cases were diagnosed in 2016, with a prediction that 14,240 people will die from RCC [1]. Recent studies on anticancer therapy targeting RCC have mainly focused on inhibitors of vascular endothelial growth factor (VEGF) and mammalian target of rapamycin (mTOR) pathways [2]. Immune checkpoint inhibitors, a prominent means of immunotherapy also known as biologic therapy, have demonstrable clinical efficacy in a subset of cancer patients [3].

Cytotoxic T-lymphocyte associated protein 4 (CTLA-4) is an essential negative regulator of T cell responses [4], resulting in T cell exhaustion and hence a state of T cell dysfunction in many types of cancer [5]. The blockade of CTLA-4 can promote anti-tumor T cell immunity [6]. In metastatic melanoma, overall survival (OS) was improved markedly with ipilimumab, a monoclonal antibody (mAb) that targets CTLA-4 and prevents B-cell activation antigen B7 and thus serving as an immune checkpoint inhibitor [7]. Nevertheless, response rates were poor, and adverse events were high, thus limiting the use of this therapy [8]. To date, only three clinical trials have assessed this anti-CTLA-4 antibody in patients with RCC [9–11]. Hence, more effective treatment strategies are required to improve therapeutic efficacy, minimizing adverse events, improving response rates, and application of immune checkpoint inhibitors.

Lycorine, a natural alkaloid derived from Amaryllidaceae plants, inhibits growth of various cancers, such as prostate cancer [12], multiple myeloma [13], ovarian cancer [14], lung epithelial cancer [15], leukemia [16–18], and melanoma [19, 20]. The possible mechanisms underlying its anticancer effect was attributed to the induction of cell apoptosis [12, 16, 18] and cell cycle arrest [13, 14, 17] and inhibition of vasculogenic mimicry formation [19]. Furthermore, a recent study demonstrated that lycorine was able to sensitize chronic lymphocytic leukemia cells to treatment in the presence of CD40L [16], highlighting that lycorine may affect the immune system. However, lycorine's anti-tumor effects in RCC have yet to be determined.

To determine the immunomodulatory potential of lycorine in RCC, we evaluated anti-tumor effects of lycorine hydrochloride alone or in combination with anti-mouse CTLA-4 in an orthotopic and metastatic tumor mice model.

## RESULTS

### Lycorine hydrochloride inhibited RCC cell viability

**Figure 1:**
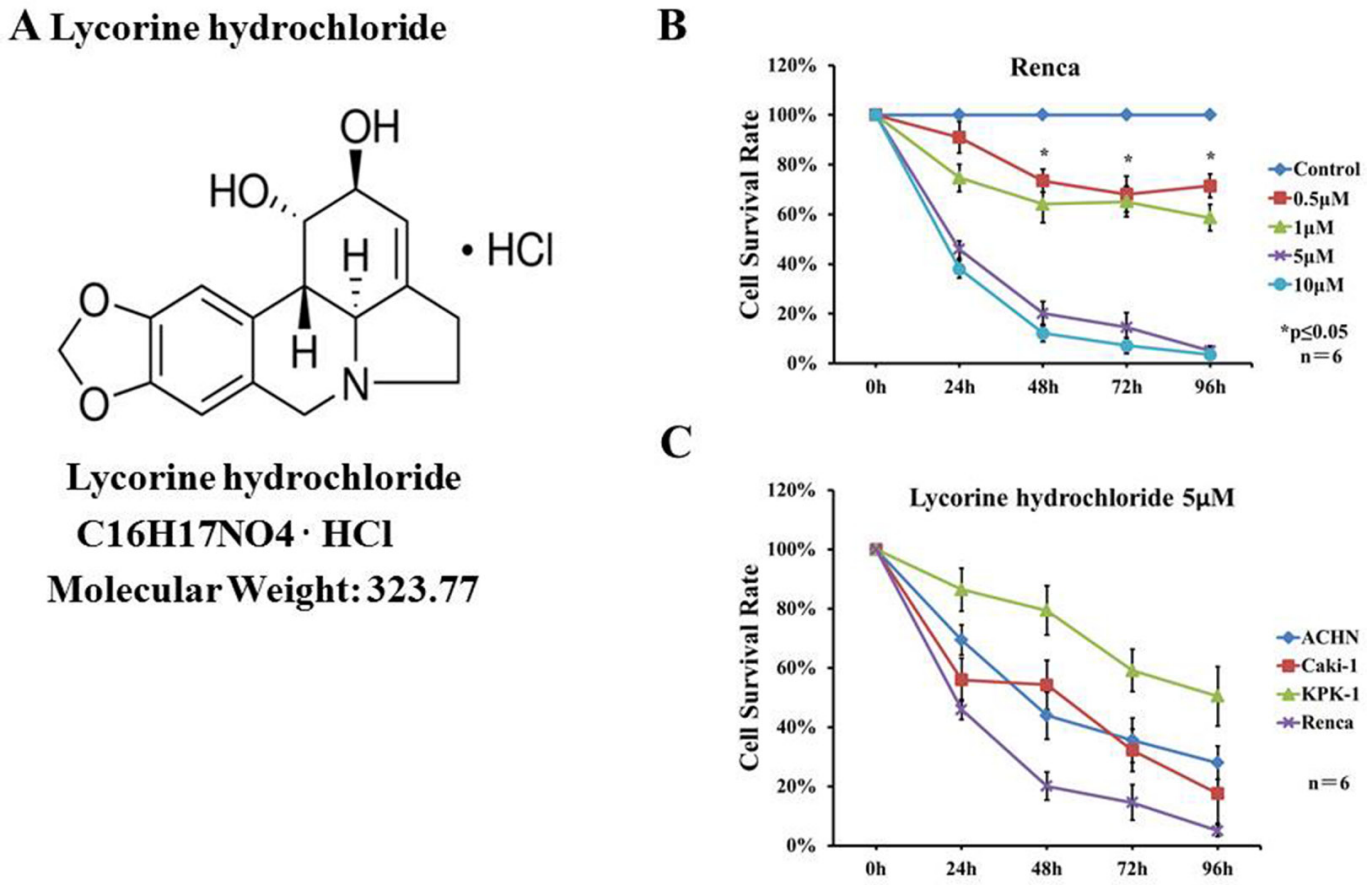
Effects of lycorine hydrochloride on the viability of renal carcinoma cells *in vitro* (**A**) The chemical structure of lycorine hydrochloride. (**B**) Renca cells were treated with serial dilutions of lycorine hydrochloride (0, 0.5, 1, 5, and 10 μM) for 96 h. Cell viability was evaluated via the XTT assay. (**C**) The XTT assay for Caki-1, ACHN, KPK-1, and Renca cells following treatment with 5 μM lycorine hydrochloride for 96 h. Each point represents the mean value ± SD. *P ≤ 0.05. Experiments were performed in triplicate.

We observed that lycorine hydrochloride inhibited Renca cell viability in a time- and dose-dependent manner (Figure 1B and Supplementary Figure 1). Additionally, lycorine hydrochloride inhibited the viability of the following RCC cell lines: Caki-1, ACHN, KPK-1, and Renca, as evaluated via an XTT assay. Here, inhibition was observed in a time-dependent inhibition manner following treatment with 5 μM lycorine hydrochloride (Figure 1C and Supplementary Figures 1, 2).

### Lycorine hydrochloride suppressed Renca migration and invasion

**Figure 2:**
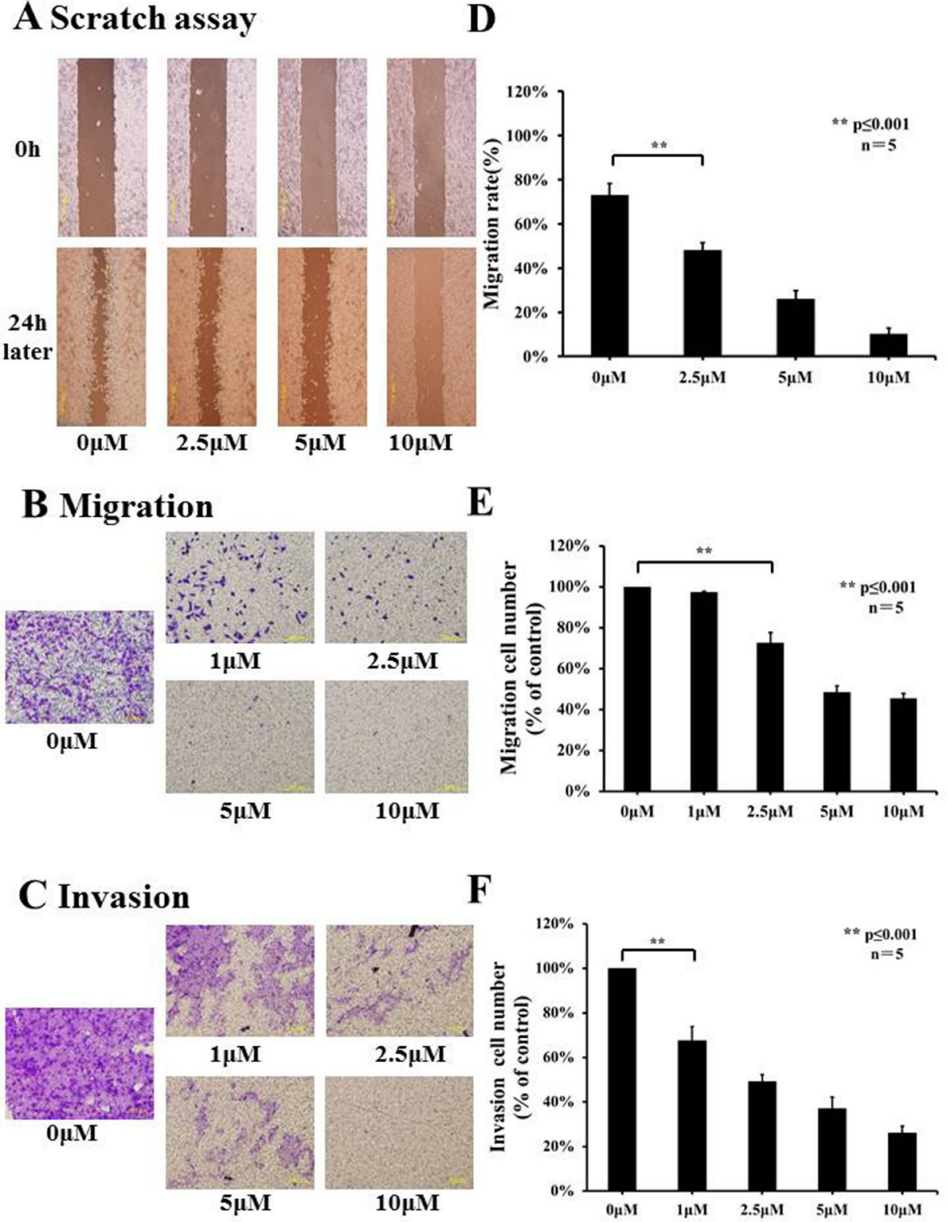
Effects of lycorine hydrochloride on migratory and invasive abilities of Renca cells *in vitro* (**A**) The effects of lycorine hydrochloride on Renca cell motility as evaluated by the scratch motility assay. Representative images of a scratch motility assay are shown with serial dilutions of lycorine hydrochloride (0, 2.5, 5, and 10 μM) after 24 h. (**D**) Cell migration was measured and results are graphically shown. Migration (**B**) and invasion (**C**) assays were used to evaluate the migratory and invasive abilities of Renca cells after treatment with serial dilutions of lycorine hydrochloride (0, 1, 2.5, 5, and 10 μM). Representative images and graph are respectively shown (**E**, **F**). Data are presented as means ± SD. **P ≤ 0.001. Scale bars: 200 μm. Experiments were performed in triplicate.

In order to determine the effects of lycorine hydrochloride on the motility of Renca cells, the scratch motility assay was performed. Lycorine hydrochloride had significant effects on Renca cell migration, with the highest concentration of lycorine hydrochloride displaying > 4-fold inhibition in the migration rate compared to the control group (10% ± 2.83% in the highest concentration group vs. 73% ± 5.15% in the control group) (Figure 2A and 2D). Subsequently, we performed migration and invasion assays to evaluate the migratory aspects of Renca cells, as shown in Figure 2B and 2C, and lycorine hydrochloride remarkably repressed the migration and invasion abilities of Renca cells.

In different dilution groups (0, 1, 2.5, 5, and 10 μM), the cell migration rates were 97.28% ± 0.65%, 72.62% ± 5.13%, 48.49% ± 3.09%, 45.34% ± 2.56% and cell invasion rates were 67.70% ± 6.18%, 49.21% ± 3.07%, 37.03% ± 5.12%, and 26.10% ± 3.08%, respectively (Figure 2E and 2F).

### Lycorine hydrochloride induced cell cycle arrest of Renca cells

**Figure 3:**
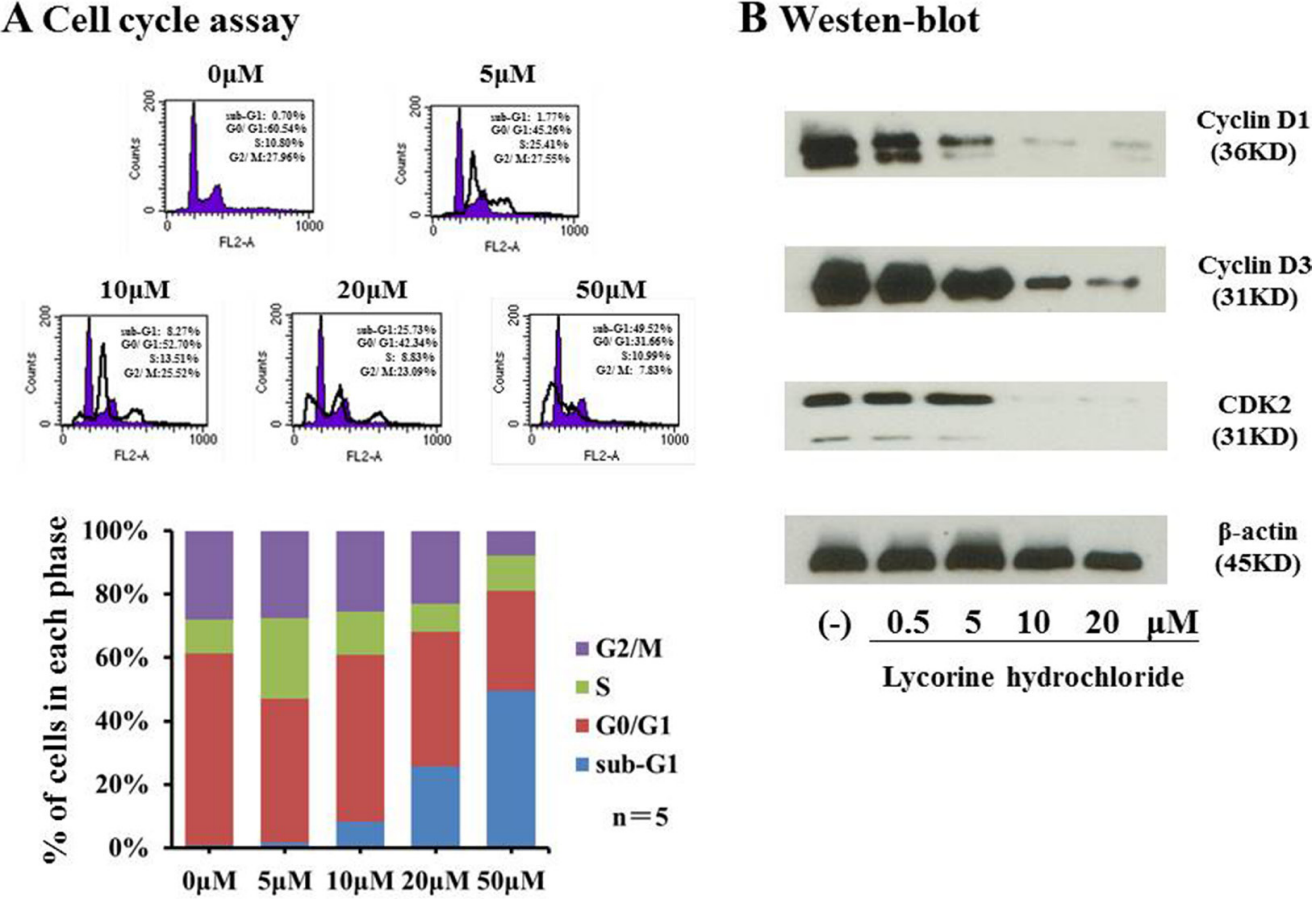
Lycorine hydrochloride arrested Renca cells (**A**) Renca cells were stained with PI and assessed via FACS after treatment with lycorine hydrochloride (0, 5, 10, 20, and 50 μM) for 12 h. Cell cycle profile is graphically shown. (**B**) Western blot analysis of cyclin D1, cyclin D3, and CDK2 expression in Renca cells after treatment with different dilutions of lycorine hydrochloride. β-actin served as the loading control. Experiments were performed in triplicate.

Flow cytometry was used to profile cell cycle dynamics, via propidium iodide (PI)/RNase Staining Buffer, upon treatment with lycorine hydrochloride. As revealed in Figure 3A, sub-G_1_ fractions (which represent the population of apoptotic cells) were markedly elevated from 1.77% to 49.52% compared with that in the control, whereas cells with difference dilutions lycorine hydrochloride treatment. By contrast, the proportions in G_2_/M cells decreased from 27.55% to 7.83% compared with that in the control. Subsequently, we performed western blot analysis to evaluate the expression of cyclin D1, cyclin D3, and CDK2 in Renca cells after treatment. As shown in Figure 3B, the expression level of all three proteins was significantly decreased as compared with that in the vehicle-treated group.

### Lycorine hydrochloride and anti-mouse CTLA-4 combination therapy led to anti-tumor effects in an orthotopic and metastatic tumor murine model

**Figure 4:**
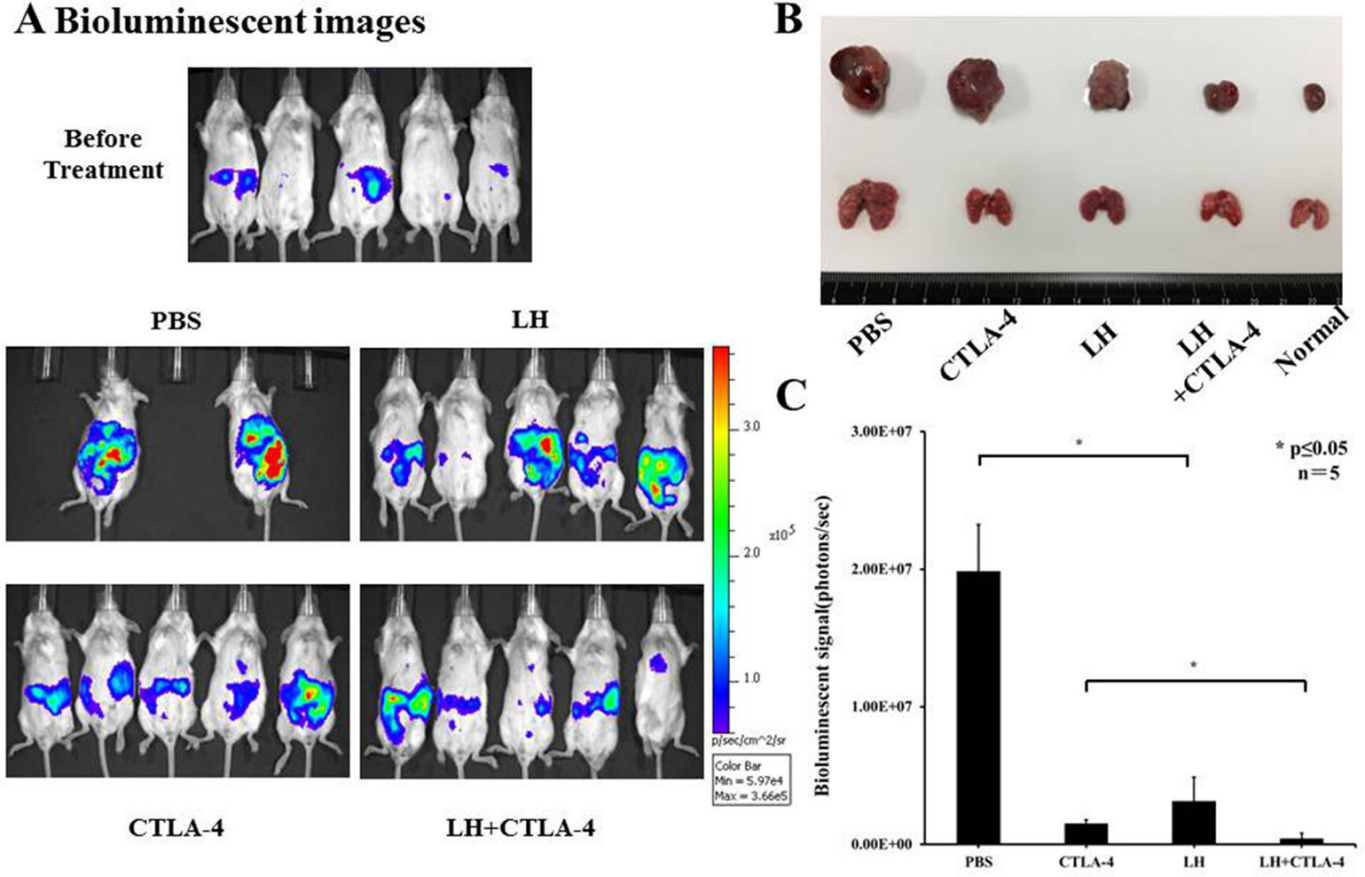
Potent anti-tumor effects of lycorine hydrochloride and anti-mouse CTLA-4 combination treatment on mice inoculated with Renca-Luc cells (**A**) Representative bioluminescence images of mice from each group are shown (day 10 post-treatment). To note, three mice in the PBS group died during treatment. Stable luciferase-Renca cells were detected using the IVIS-200 Imaging System. (**C**) Each tumor volume was detected via the IVIS-200 Imaging System. Data were analyzed among groups and significant differences were observed in the lycorine hydrochloride and anti-mouse CTLA-4 combination treatment group (*N* = 5). (**B**) Representative macroscopic images of the orthotopic and metastatic tumor were measured among groups and significant differences were observed in mice administered the combination treatment. All data are presented as means ± SD. *P ≤ 0.05. Experiments were done in triplicate.

To appraise *in vivo* effects of lycorine hydrochloride and anti-mouse CTLA-4 combination treatment, mice were randomly divided into four groups and received treatment as outlined in the Materials and Methods. Tumor volumes were detected by the IVIS-200 Imaging System on day 10 post-treatment (Figure 4A). The photo flux indices (Figure 4C) were analyzed among groups, and remarkable, significant differences were observed in the combination treatment group as compared with other groups. Moreover, macroscopic images of the orthotopic and metastatic tumors were captured (Figure 4B) via the IVIS instrument.

### Lycorine hydrochloride and anti-mouse CTLA-4 combination therapy suppressed T_regs_ and upregulated effector T cells

**Figure 5:**
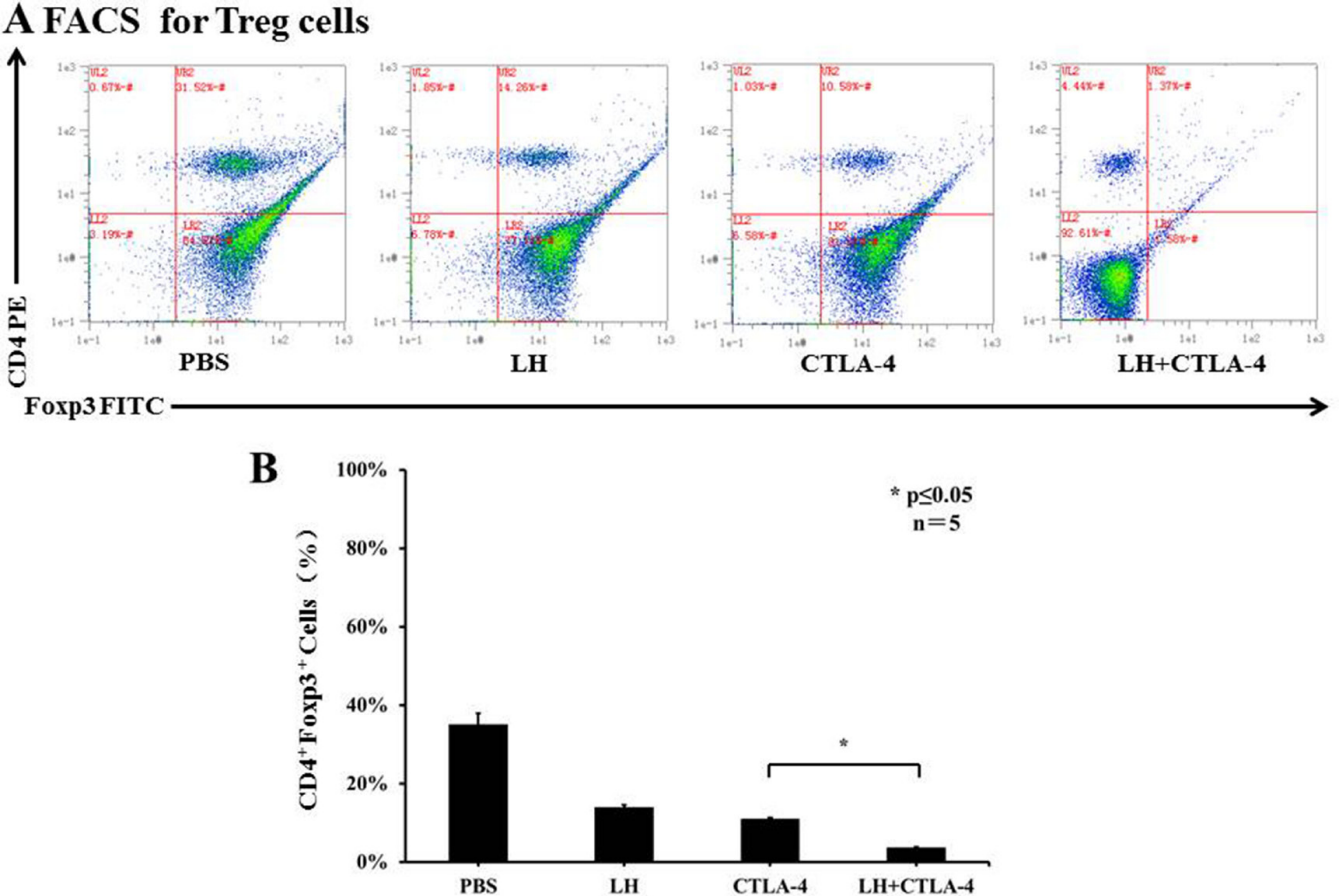
The frequency of CD4+ Foxp3+ T_regs_ in peripheral blood (**A**) Blood samples from mice (control and treatment groups) were stained with CD4 and Foxp3 and analyzed by flow cytometry. Representative data are shown. (**B**) Results are graphically shown with significant differences observed in the lycorine hydrochloride and anti-mouse CTLA-4 combination treatment group compared to the other groups. Data are presented as means ± SD. *P ≤ 0.05. Experiments were done in triplicate.

**Figure 6:**
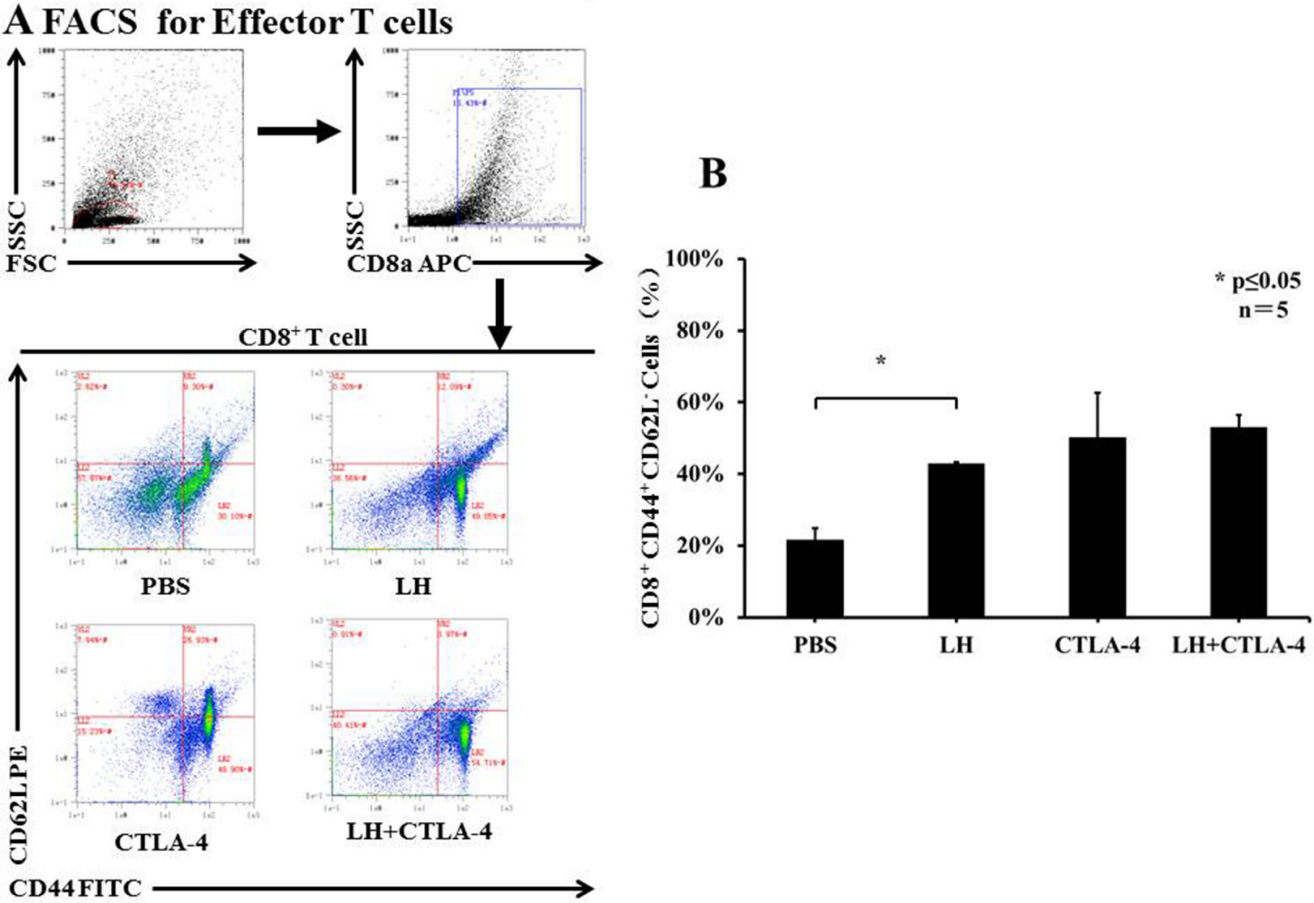
The frequency of CD8+ CD44+ CD62L− effector T cells in peripheral blood (**A**) Blood samples from mice (control and treatment groups) were stained with CD8, CD62L, and CD44 and analyzed by flow cytometry. Representative data are shown. (**B**) Results are graphically shown and significant differences were observed in the treatment groups in comparison with the control group. Data are presented as means ± SD. *P ≤ 0.05. Experiments were done in triplicate.

T-regulatory cells (T_regs_) are known to suppress immune responses towards tumors. The frequency of CD4+ Foxp3+ T_regs_ was higher in control, tumor bearing BALB/c mice, accounting for approximately 35% of total splenocytes, but there was a significant decrease of T_regs_ in the combination group as compared with the other treatment groups (3.70 ± 0.23% in combination group vs. 13.88 ± 0.75% in lycorine hydrochloride group and 10.98 ± 0.38% in anti-mouse CTLA-4 group) (Figure 5A and 5B). In addition, the frequency of CD8+ CD44+ CD62L− effector T cells was assessed by flow cytometry. Intriguingly, we observed a remarkable upregulation of effector T cells in the treatment groups: 52.95 ± 3.40% in the combination group, 42.89 ± 0.49% in the lycorine hydrochloride group, and 50.15 ± 12.53% in the anti-mouse CTLA-4 group, respectively, compared to the control group (Figure 6A and 6B). Furthermore, histopathological examination indicated no abnormal histological changes in any treatment group (data not shown).

## DISCUSSION

RCC management has undergone a major transformation over the past decade [21]. Innovative surgical strategies such as partial nephrectomy can provide excellent oncological outcomes compared with radical nephrectomy in localized disease [22]. The European Association of Urology (EAU) Guidelines also recommend cytoreductive nephrectomy and VEGF- and mTOR-targeted therapies for patients with metastatic RCC (mRCC) [22].

Immunotherapy, agents such as IL-2 (interleukin-2) and interferon have been used for more than 20 years. In addition, novel agents, such as immune checkpoint inhibitors CTLA-4 blockage, PD-1 and PD-L1 blockage, are currently undergoing clinical trials and show promising results [23]. Two phase III studies are investigating PD-1, CTLA-4, and PD-L1 antibodies in combination with anti-VEGF therapy for patients with mRCC (http://www.clinicaltrials.gov/ct2/show/NCT02231749, NCT02420821). Nevertheless, the effectiveness of immune checkpoint inhibitors in patients with mRCC is very low. Therefore, developing a new therapeutic strategy with improved effectiveness is of paramount importance. Lycorine has previously been suggested to exhibit potential anti-cancer effects [12–19]. Furthermore, according to the previous study, the activation of lycorine was associated with the immune system [16]. More recently, we demonstrated that a combination of survivin inhibitor and immunotherapy exhibited a potent therapeutic effect in RCC [24].

In the current study, we hypothesized that combining lycorine hydrochloride with emerging immunotherapeutic agent anti-CTLA-4 mAb could result in a potentially more effective anticancer strategy. The inhibition of cell proliferation was measured using IC_50_ values (Supplementary Figure 1). We clearly confirmed the anticancer potential of lycorine hydrochloride *in vitro* as we observed time-dependent inhibition in several RCC cell lines, particularly following treatment with 5 μM lycorine hydrochloride (Figure 1B and Supplementary Figure 2), and the Renca cell line was the most sensitive to the treatment (Figure 1C and Supplementary Figure 1). Moreover, lycorine hydrochloride suppressed the migratory and invasive abilities of Renca cells (Figure 2). Previous studies indicated that there is a strong relationship between cell cycle arrest and the anticancer effect of lycorine [13, 14, 17, 20, 25, 26].

To further investigate the possible mechanism underlying the anticancer potential of lycorine hydrochloride, we discerned its effect on the cell cycle profile and found that sub-G_1_ fractions and G_2_/M DNA content respectively increased and decreased after treatment (Figure 3A). Compared with the control group, the sub-G_1_ phase cell proportion increased by approximately 48.82% and G_2_/M cells proportion decreased by 20.13% in the highest concentration group. The expression of cyclin D1, cyclin D3, and CDK2 was also significantly decreased upon lycorine treatment (Figure 3B). A previous study has demonstrated significant associations between cell cycle arrest and cyclin D1, cyclin D3, and CDK2 depletion [27], which may partly explain their anticancer effect in RCC. We then investigated whether the combination of lycorine hydrochloride and anti-mouse CTLA-4 would treat RCC and mRCC, one of the most common malignant tumors. In our study, when compared to mice treated only with anti-CTLA-4 or lycorine hydrochloride therapy, mice on the combination treatment regimen showed significantly reduced orthotopic and metastatic tumors (Figure 4). In addition, we observed a synergistic effect of immunological suppression using this combination treatment strategy, which may partially explain the robust anti-tumor effects of this treatment regimen.

Herein, for the first time, we report that lycorine hydrochloride in combination with anti-mouse CTLA-4 inhibited orthotopic and metastatic tumors by downregulating T_regs_ (Figure 5), which was accompanied with upregulation of effector T cells (Figure 6). Our findings suggest and indicate lycorine as a potent candidate for treating RCC. Moreover, our study demonstrated that lycorine functions partially via immunological activation as per the observed dynamics of T_regs_ and effector T cells. Importantly, we have highlighted the synergistic effect of immunological suppression in the combination treatment group, and such insight implies that lycorine may partly enhance patient immune response rates. In summary, the effect of combined administration of lycorine hydrochloride and anti-mouse CTLA-4 exhibited a potent therapeutic effect in the orthotopic and metastatic RCC tumor murine model and will serve as an excellent aid for developing a better treatment strategy for RCC.

However, this study has some limitations, e.g., the exact molecular target of anticancer effect on RCC has not yet been identified. Thus, more research needs to be conducted in the future.

## MATERIALS AND METHODS

### Cells and cell culture

Caki-1; ACHN; KPK-1, the human adenocarcinoma cell lines; and Renca, a murine kidney carcinoma cell line, were purchased from the American Type Culture Collection (ATCC, Rockville, MD, USA) and cultured at 37°C and 5% CO_2_. The cells were maintained in Roswell Park Memorial Institute (RPMI) 1640 Medium supplemented with 10% fetal bovine serum (Gibco, Invitrogen, Carlsbad, CA, USA).

### Animals

BALB/c mice (female, 8 weeks old, 20 ± 2 g) were purchased from Japan SLC, Inc. (Hamamatsu, Japan) and used for this study. All mice were bred and housed in a specific pathogen-free (SPF) environment at the Department of Animal Center of Okayama University (Okayama, Japan). To allow adequate acclimation, mice were housed in the SPF environment for more than 1 week prior to experimentation. Animals were bred and handled in accordance with the guidelines of the Okayama University Animal Research Committee.

### Drugs and antibodies

Lycorine hydrochloride (Figure 1A) was obtained from Sigma-Aldrich, Inc. (St. Louis, MO, USA). The anti-mouse CTLA-4 (clone 9H10) monoclonal antibody was purchased from BioXCell (West Lebanon, NH, USA). Primary antibodies against cyclin D1, cyclin D3, CDK2, and β-actin were purchased from Cell Signaling Technology, Inc. (Danvers, MA, USA). Purified PE rat anti-mouse CD4, FITC rat anti-mouse Foxp3, APC rat anti-mouse CD8a, PE rat anti-mouse CD62L, and FITC rat anti-mouse CD44 were purchased from BD Biosciences, Inc. (San Jose, CA, USA).

### XTT assay

The Cell Proliferation Kit II (Roche Diagnostics, Indianapolis, IN, USA) was used to assess the effects of lycorine hydrochloride on cell viability. Briefly, the cell lines Caki-1; ACHN; KPK-1 and Renca were diluted in 96-well plates (Costar, Corning, NY, USA) at a density of 1 × 10^3^ and incubated with medium alone or serial dilutions of lycorine hydrochloride (0, 0.5, 1, 5, and 10 μM). Cell viability was monitored after 24, 48, 72, and 96 h. For each time point, the XTT labeling reagent and an electron coupling reagent was added to the culture. The cells were incubated for 4 h at 37°C and 5% CO_2_, and absorbance at 450–500 nm, with a reference wavelength at 650 nm, was recorded using a Bio-Rad microplate reader (Hercules, CA, USA).

### Scratch motility assay

Renca cell migratory ability was evaluated via the scratch motility assay. Cells were seeded in 6-well plates (Costar, Corning, NY, USA) at a density of 3 × 10^5^ for 24 h. A vertical wound was made with a sterile pipette tip, followed by washing with PBS two times, and then incubated with medium alone or serial dilutions of lycorine hydrochloride (0, 2.5, 5, and 10 μM) for 24 h at 37°C and 5% CO_2_. Images of cell migration onto these scratched zones were captured by the Olympus IX2 microscope (Olympus, TOKYO, JAPAN). The scratch width was calculated using the Image Pro Plus 6.0 software. Migration rate was calculated as (%) = [1 −*W (24 h)]/*W (0 h), where *W (0 h) and *W (24 h) indicate the width of the wound at 0 h and 24 h, respectively.

### Migration and invasion assays

For the migration assay, Renca cells were plated in six-well plates at a density of 3 × 10^5^ and incubated with medium alone or serial dilutions of lycorine hydrochloride (0, 1, 2.5, 5, and 10 μM) for 24 h. Renca cells were resuspended in serum-free medium and seeded into trans-well chambers with 8-μm diameter pore size polycarbonate membranes (Corning, NY, USA). Medium containing 30% fetal bovine serum was used in the lower chamber as an attractant. For the invasion assay, trans-well chambers were coated with Corning Matrigel Matrix (Corning, NY, USA). After 12 h, cells on the upper chamber surface were removed using cotton swabs. The migrated or invaded cells in the lower surface were fixed with 4% paraformaldehyde and stained with 0.1% crystal violet. The total numbers of cells were captured and analyzed from 10 different fields with the Olympus IX2 microscope.

### Flow cytometry

For *in vitro* assays, Renca cells were plated onto 6-well plates at a density of 3 × 10^5^ and incubated with medium alone or serial dilutions of lycorine hydrochloride. Cells were washed with PBS after 12 h, fixed with 75% ethanol, and further incubated at 4°C for 2 h. Then, cells were resuspended with propidium iodide (PI)/RNase Buffer (BD Biosciences, San Jose, CA, USA). After being incubated in the dark for 15 min at 4°C, flow cytometric analyses were performed and analyzed via MACSQuant Analyzer 10 (Miltenyi Biotec, San Diego, CA, USA). For *in vivo* assays, blood samples from mice were collected into tubes containing ethylenediaminetetraacetic acid (EDTA). Samples were then incubated with the appropriate antibody for 1 h at 4°C and washed twice with PBS. For the intracellular staining of Foxp3, cells were fixed and permeabilized as previously described [28]. Samples were resuspended in 250 μL of cold PBS and analyzed using the MACSQuant Analyzer 10.

### Western blot analysis

Western blot analyses were performed as previously described [28]. A total of 20 μg of protein was separated in 10% sodium dodecyl sulfate (SDS)-polyacrylamide gels and transferred to polyvinylidene difluoride (PVDF) membranes (Bio-Rad, Hercules, CA, USA). The PVDF membranes were incubated with the indicated primary antibodies overnight at 4°C after being blocked with 5% nonfat dry milk in 1 X TBS-T (0.05 M Tris-buffered saline with 0.5% Triton X-100). After washed three times with 1 X TBS-T, the membranes were incubated with anti-rabbit or anti-mouse horseradish peroxide secondary antibodies, and protein abundance was assessed via exposing the membranes to X-ray film after incubation with an enhanced chemiluminescence reagent (ECL kit; Amersham Pharmacia Biotech, Chandler, AZ, USA).

### Bioluminescent imaging of tumors in live mice

Luciferase-expressing tumors were detected by IVIS-200 Imaging System (Xenogen Corporation, Alameda, CA, USA). Briefly, mice were injected by intraperitoneally (IP) with D-luciferin (150 mg/kg, Thermo Fisher Scientific, Waltham, MA, USA) after being anesthetized. Images were acquired with the IVIS-200 Imaging System at appropriate exposure times, the imaging and quantification of signals were analyzed by the Living Image software with proper background subtraction.

### *In vivo* lycorine hydrochloride and anti-mouse CTLA-4 combination treatment

For our *in vivo* therapeutic experiments, Renca-Luc cells were inoculated into the sub-renal capsule kidney (3 × 10^5^ cells in 50 μl PBS) and tail vein (2 × 10^5^ cells in 30 μl PBS) of C57BL/6 mice to produce an orthotopic tumor and metastatic lung tumors. Mice were randomly divided into four groups: PBS (control), lycorine hydrochloride, anti-mouse CTLA-4, and the combination (lycorine hydrochloride plus anti-mouse CTLA-4), a week later in accordance with the bioluminescent imaging captured by the IVIS-200 Imaging System. Briefly, mice received an intraperitoneal (IP) injection of 200 μL of PBS as a vehicle control, lycorine hydrochloride (5 mg/kg per day for a week) and/or anti-mouse CTLA-4 (100 μg/mouse on day 0 and 7 of treatment). The bioluminescent imaging and tumor volume was analyzed on day 10 post-treatment. Mice were then sacrificed, and the macroscopic images of orthotopic and metastatic tumors were detected.

### Statistical analysis

All data are expressed as mean values ± standard deviation (SD), except cell cycle data, which are presented as means. All statistical were analyzed with SPSS 20.0 software package (SPSS Inc., Chicago, IL, USA). One-way ANOVA, followed by Bonferroni post-hoc comparison tests (in case of equal variances) or Welch and Brown-Forsythe tests (in case of unequal variances), was performed for comparisons among control and treatment groups. *P* ≤ 0.05 was considered statistically significant.

## SUPPLEMENTARY MATERIALS FIGURES AND TABLES


