# Supplementary material for: Synergistic effects of the immune checkpoint inhibitor CTLA-4 combined with the growth inhibitor lycorine in a mouse model of renal cell carcinoma
Source: Oncotarget. 2017 Feb 19;8(13):21177–86. doi: 10.18632/oncotarget.15505 (PMC5400575; doi:10.18632/oncotarget.15505)
Supplement: Supplementary file 1 [file oncotarget-08-21177-s001.pdf]

Synergistic effects of the immune checkpoint inhibitor CTLA-4 combined with the growth inhibitor lycorine in a mouse model of renal cell carcinoma

Supplementary Materials

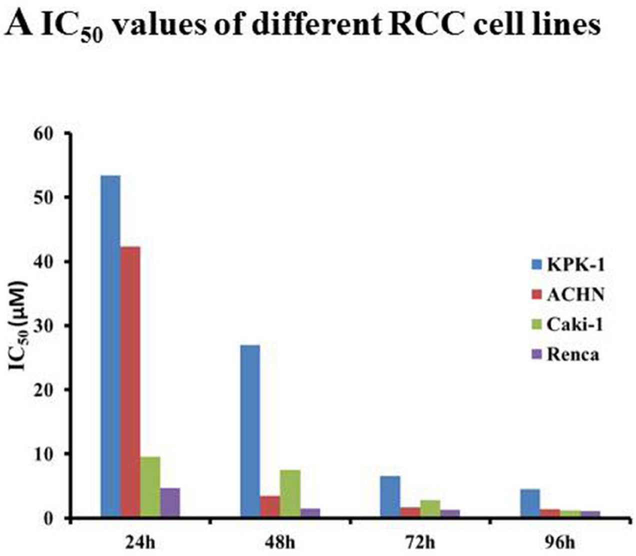

B

KPK-1

24h	53.364 μM
48h	26.941 μM
72h	6.571 μM
96h	4.517 μM

ACHN

24h	42.311 μM
48h	3.504 μM
72h	1.662 μM
96h	1.360 μM

Caki-1

24h	9.565 μM
48h	7.498 μM
72h	2.819 μM
96h	1.202 μM

Renca

24h	4.701 μM
48h	1.506 μM
72h	1.255 μM
96h	1.068 μM

Supplementary Figure 1: IC_{50} values of different RCC cell lines. The XTT assay for Caki-1, ACHN, KPK-1, and Renca cells following treatment with serial dilutions of lycorine hydrochloride (0, 0.5, 1, 5, and 10 μM) for 96 h. Average IC50 values at several time points (24, 48, 72 and 96 h) were plotted (**A**, **B**). Experiments were performed in triplicate.

MTT assay of different RCC cell lines

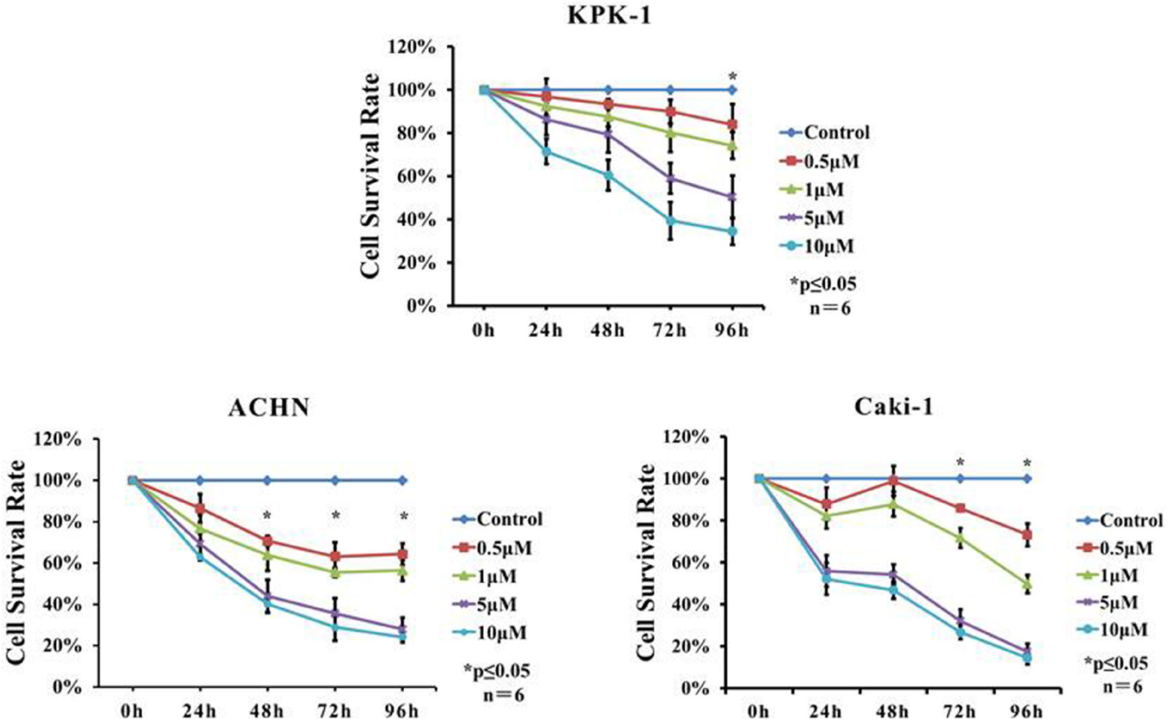

Supplementary Figure 2: Effects of lycorine hydrochloride on the viability of Caki-1, ACHN, KPK-1 cells *in vitro*. Caki-1, ACHN and KPK-1 cells were treated with serial dilutions of lycorine hydrochloride (0, 0.5, 1, 5, and 10 μM) for 96 h. Cell viability was evaluated via the XTT assay. Each point represents the mean value ± SD. *$P \leq 0.05$. Experiments were performed in triplicate.
